# Psoralen alleviates radiation-induced bone injury by rescuing skeletal stem cell stemness through AKT-mediated upregulation of GSK-3β and NRF2

**DOI:** 10.1186/s13287-022-02911-2

**Published:** 2022-06-07

**Authors:** Bo-Feng Yin, Zhi-Ling Li, Zi-Qiao Yan, Zheng Guo, Jia-Wu Liang, Qian Wang, Zhi-Dong Zhao, Pei-Lin Li, Rui-Cong Hao, Meng-Yue Han, Xiao-Tong Li, Ning Mao, Li Ding, Da-Fu Chen, Yue Gao, Heng Zhu

**Affiliations:** 1 Beijing Institute of Radiation Medicine, Road Taiping 27, Beijing, 100850 People’s Republic of China; 2 Beijing Key Laboratory for Radiobiology, Beijing Institute of Radiation Medicine, Beijing, 100850 People’s Republic of China; 3 People’s Liberation Army General Hospital, Road Fuxing 28, Beijing, 100853 People’s Republic of China; 4 Medical Center of Air Forces, PLA, Road Fucheng 30, Beijing, 100142 People’s Republic of China; 5 Graduate School of Anhui Medical University, 81 Meishan Road, Shushan Qu, Hefei, 230032 Anhui People’s Republic of China; 6 Beijing Institute of Basic Medical Sciences, Road Taiping 27, Beijing, 100850 People’s Republic of China; 7 Laboratory of Bone Tissue Engineering, Beijing Laboratory of Biomedical Materials, Beijing Research Institute of Traumatology and Orthopaedics, Beijing Jishuitan Hospital, Eastern Street Xinjiekou 31, Beijing, 100035 China

**Keywords:** Psoralen, Radiation-induced bone injuries, Skeletal stem cells, NRF2

## Abstract

**Background:**

Repairing radiation-induced bone injuries remains a significant challenge in the clinic, and few effective medicines are currently available. Psoralen is a principal bioactive component of *Cullen corylifolium* (L.) Medik and has been reported to have antitumor, anti-inflammatory, and pro-osteogenesis activities. However, less information is available regarding the role of psoralen in the treatment of radiation-induced bone injury. In this study, we explored the modulatory effects of psoralen on skeletal stem cells and their protective effects on radiation-induced bone injuries.

**Methods:**

The protective effects of psoralen on radiation-induced osteoporosis and irradiated bone defects were evaluated by microCT and pathological analysis. In addition, the cell proliferation, osteogenesis, and self-renewal of SSCs were explored. Further, the underlying mechanisms of the protective of psoralen were investigated by using RNA sequencing and functional gain and loss experiments in vitro and in vivo. Statistical significance was analyzed using Student's t test. The one-way ANOVA was used in multiple group data analysis.

**Results:**

Here, we demonstrated that psoralen, a natural herbal extract, mitigated radiation-induced bone injury (irradiation-induced osteoporosis and irradiated bone defects) in mice partially by rescuing the stemness of irradiated skeletal stem cells. Mechanistically, psoralen restored the stemness of skeletal stem cells by alleviating the radiation-induced suppression of AKT/GSK-3β and elevating NRF2 expression in skeletal stem cells. Furthermore, the expression of KEAP1 in skeletal stem cells did not significantly change in the presence of psoralen. Moreover, blockade of NRF2 in vivo partially abolished the promising effects of psoralen in a murine model of irradiation-induced osteoporosis and irradiated bone regeneration.

**Conclusions:**

In summary, our findings identified psoralen as a potential medicine to mitigate bone radiation injury. In addition, skeletal stem cells and AKT-GSK-3β and NRF2 may thus represent therapeutic targets for treating radiation-induced bone injury.

**Graphical Abstract:**

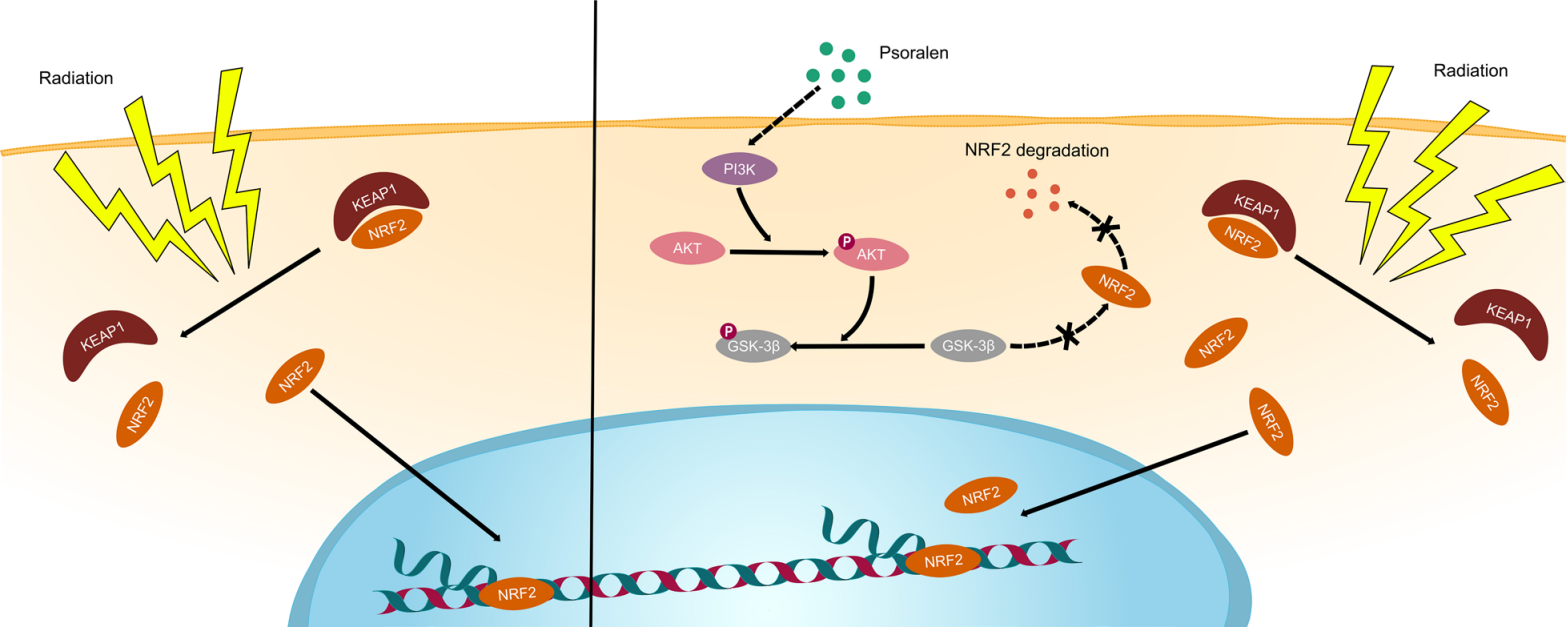

**Supplementary Information:**

The online version contains supplementary material available at 10.1186/s13287-022-02911-2.

## Introduction

Radiation-induced bone injury during cancer therapy remains a significant challenge in clinical trials [1, 2]. To resolve skeletal tumors, the combination of surgical bone removal and radiation therapy usually results in large bone defects and hampers bone regeneration [3, 4]. In addition, radiation therapy increases the risks of osteoporosis and fracture in non-skeletal cancer patients [5, 6]. However, the cellular and molecular mechanisms of radiation-induced bone injury are not completely understood, which greatly hinders the development of effective medicines and novel strategies to treat radiation-induced bone injury.

Skeletal stem cells (SSCs) were recently identified as the endogenous stem cells of skeletal tissues [7–10]. The pioneering works of Chan et al. and Worthley et al. demonstrated that numerous subpopulations of SSCs remained in murine bones and contributed to bone formation [7, 8]. The identification of SSCs in human bones further highlights the contributions of SSCs to skeletal regeneration [9]. Moreover, our most recent study dissected the ontogeny of human embryonic skeletal stem cells at the single-cell level [10]. Although increasing data have demonstrated that SSCs are closely involved in skeletal development, maintenance, and regeneration, less information is available regarding the role of SSCs in the treatment of radiation-induced bone injury. Chandra et al. reported that the suppression of sclerostin alleviated radiation-induced bone loss in a focal irradiation model by protecting bone-forming cells and their progenitors [11]. Our previous work showed that stimulation with ferulic acid promoted the repair of irradiated bone defects by protecting SSCs [12]. These findings suggest that endogenous and exogenous SSCs may be potential targets and tools for the treatment of radiation-induced bone injury.

*Cullen corylifolium* (L.) Medik has long been considered a potent Chinese traditional medicine for skeleton healthcare. Psoralen is a principal bioactive component of *Cullen corylifolium* (L.) Medik and has been reported to have antitumor, antiviral, antibacterial, and anti-inflammatory activities [13–15]. Notably, previous studies have demonstrated that psoralen increases the gene expression levels of osteoblast-specific markers, including Runx2, Col-I, and Sp7, and enhances osteoblast differentiation. Moreover, psoralen inhibits osteoclast differentiation and is an essential regulator of osteoblast and osteoclast function in tumor-bearing mice [16–18]. However, it is unknown whether psoralen protects skeletal tissue from radiation injury.

Nuclear factor (erythroid-derived 2)-like 2 (Nfe2l2, NRF2) is one of the basic leucine zipper transcription factors that binds to the regulatory regions of genes encoding antioxidant proteins and detoxifying enzymes [19, 20]. Kelch-like ECH-associated protein 1 (KEAP1) forms a ubiquitin E3 ligase complex that promotes NRF2 degradation and controls NRF2 nuclear levels. In addition to the regulation of antioxidant activity, NRF2 has been previously reported to have a role in controlling bone homeostasis [21, 22]. However, previous studies have yielded controversial results partially due to the global genetic modifications of Nfe2l2 in mice affecting all cell types and tissues [23, 24]. Thus, cell-type specific studies of NRF2 may be helpful to understand its role in bone biology and pathophysiology.

Based on the emerging role of SSCs in the repair of bone radiation injury and the potential role of psoralen in the treatment of bone disorders, we explored the rescuing effects of psoralen on irradiation-induced osteoporosis and irradiated bone defects in mice. Moreover, the cellular and molecular mechanisms underlying the protective effects of psoralen on bone radiation injury were also investigated.

## Materials and methods

### Animals

Eight-week-old female C57BL/6 N mice and 1-week-old female C57BL/6 N mice were purchased from Beijing Vital River Laboratory Animal Technology Co., Ltd. All of the animal experiments were performed in accordance with the Academy of Medical Sciences Guide for Laboratory Animals (IACUC-DWZX-2021-654).

### Psoralen preparation

Psoralen (98% purity, CAS 66-97-7) was purchased from EFE BIO (Shanghai, China), dissolved in DMSO to prepare a 13.429 mM as a stock solution and then diluted in cell culture medium so that DMSO comprised < 0.1% of the total volume. The optimized concentrations of psoralen were screened by Cell Counting Kit-8 (CCK-8) assay with graded concentrations of psoralen (0, 1, 5, 10, 15, and 20 μM). The optimal concentration of psoralen was determined to be 10 μM. This concentration was used in the experiments unless otherwise specified. In some experiments, the SSCs were pretreated with psoralen for 48 h before usage.

### Cell culture

Murine SSCs were isolated from lone bones according to our previously reported procedure with minor revisions [12, 25, 26]. In the present study, the metaphysis was maintained because it has been proved as a pool of SSCs which showed stronger osteochondral potential than that of mesenchymal stem cells [7, 8]. Briefly, femurs and tibiae from 1-week-old C57BL/6 N mice were dissected, and the bone marrow cells were flushed out before metaphyses and diaphyses were chopped and digested by collagenase II (Sigma-Aldrich, 0.1% vol/vol) at 37 °C for 1 h. The released cells were discarded, and the digested bone chips were cultured in minimum essential medium eagle, alpha modification (α-MEM; Invitrogen, Carlsbad, CA) supplemented with 10% fetal bovine serum (FBS) (HyClone, Logan, UT). The adherent cells were cultured in a 37 °C incubator with 5% CO_2_. Cells from passages 3–6 were used for in vivo and in vitro experiments unless otherwise described. The PI3K/AKT inhibitor LY294002 (MCE, China) and NRF2 inhibitor ML385 (APExBIO, USA) were dissolved in DMSO to prepare 10 mM stock solutions and then diluted in cell culture medium to the appropriate experimental concentrations.

### Cell toxicity and proliferation

The effect of psoralen on the proliferation of SSCs/irradiated SSCs was assessed using a cell viability assay (Cell Counting Kit-8 (CCK-8); Dojindo, Japan). Briefly, SSCs were seeded in 96-well plates at an initial density of 1 × 10^3^ cells/well and cultured with different concentrations of psoralen (0, 1, 5, 10, 15, and 20 μM) for 1, 3, 5, and 7 days. Then, 10 μL of CCK-8 solution and 90 μL of culture medium were added to each well at each time point and incubated for 1 h at 37 °C. The absorbance of each sample was measured at 450 nm.

### Colony-forming unit fibroblast (CFU-F) assay

In the current study, SSC self-renewal was assessed with CFU-F assay. SSCs/irradiated SSCs were seeded in triplicate into six-well plates (5 × 10^2^ cells/well) and cultured for up to 14 days until colonies were clearly visible. Colonies were fixed with 4% paraformaldehyde and stained with 0.5% crystal violet, and colonies with more than 50 cells were counted. Psoralen was added at a gradient of concentrations to investigate the effects of psoralen on the self-renewal of SSCs. Three independent experiments were performed for each assay.

### Osteogenic induction and ALP and von Kossa staining

According to our previous study [12, 25], in vitro osteogenesis of SSCs and irradiated SSCs was investigated by seeding cells (5 × 10^3^/well) into 48-well plates (five wells in each group) and induced using osteogenic differentiation medium containing 100 nM dexamethasone, 10 mM β-glycerophosphate, and 50 μg/ml ascorbic acid (all from Sigma-Aldrich) supplemented or not with 10 μM psoralen.

For ALP staining, cells were assayed using an Alkaline Phosphatase Kit following the manufacturer’s instructions after 14 days of differentiation. For von Kossa staining, cells were assayed after 28 days of differentiation. Briefly, cells were fixed with 4% paraformaldehyde for 30 min. After washing the cells with distilled water three times, the cells were stained with 5% silver nitrate water solution and incubated under bright sunlight for 30 min. The cells were washed again, the water was removed, and a 5% sodium thiosulfate water solution was added for 5 min.

### RNA sequencing analyses of SSCs

To further investigate the underlying mechanism of irradiation-mediated impairment and the underlying mechanism of psoralen treatment on SSCs, SSCs isolated from normal/irradiated mice (2 Gy, 1 h after irradiation) and SSCs cultured or not with 10 μM psoralen for 24 h were subjected to high-throughput RNA sequencing analyses. Next-generation sequencing library preparations and Illumina MiSeq sequencing were conducted at GENEWIZ, Inc. (Suzhou, China). DNA libraries were validated with an Agilent 2100 bioanalyzer (Agilent Technologies, Palo Alto, CA, USA) and quantified with a Qubit 2.0 Fluorometer. DNA libraries were multiplexed and loaded onto an Illumina MiSeq instrument per the manufacturer's instructions (Illumina, San Diego, CA, USA). Sequencing was performed using a 2 × 300 paired-end (PE) configuration; image analysis and base calling were conducted by MiSeq Control Software (MCS) embedded in the MiSeq instrument. All differentially abundant mRNAs were used for GO analysis to deepen the understanding of the molecular mechanism of the cell biological information processes.

### Animal model of radiation-induced bone injuries

To study the effect of psoralen on radiation-induced osteoporosis, C57BL/6 N mice were randomly divided into four groups (normal, radiation, prevention, and treatment). The dosage of psoralen administered (20 mg/Kg) to the model animals was determined based on the literature [27]. For the prevention/treatment group, C57BL/6 N mice were administered psoralen (20 mg/Kg) intragastrically every day for 1 week before/after radiation and killed 1 week after radiation. The mice were exposed to a 2 Gy of a whole-body dose of gamma-radiation (Co-60) at a rate of 0.98 Gy/min. ML385 (30 mg/Kg) was injected intraperitoneally 1 h before psoralen treatment [28]. To study the effect of psoralen on SSCs in the irradiated bone defect model, only mouse femurs (the other parts covered with a lead board) were exposed to a 2 Gy dose of gamma-radiation (Co-60) at a rate of 0.98 Gy/min under anesthesia. Bone defect surgeries were conducted immediately after irradiation. A 1.0 mm circular defect was generated in the right distal femur close to the metaphysis. Mice were killed at 1, 2, and 3 weeks post-surgery. Upon specimen harvest, femoral samples were fixed in 4% paraformaldehyde for 48 h at 4 °C, transferred into 70% ethanol, and stored at 4 °C for further experiments.

### SSC/irradiated SSC transplantation

Adherent cells were exposed to a 2 Gy dose of gamma-radiation (Co-60) at a rate of 0.98 Gy/min for the following in vivo and in vitro experiments. To facilitate SSC transplantation, SSC microcryogels were prepared according to previous reports [29, 30]. Biodegradable gelatin microcryogels were obtained from Beijing Cytoniche Co., Ltd. (http://www.cytoniche.com/). Briefly, normal/irradiated SSCs or psoralen-pretreated normal/irradiated SSCs (48 h before surgery) were harvested and resuspended at a concentration of 1 × 10^7^ cells/ml in PBS (5% fetal bovine serum). From each of these suspensions, 200 μL was pipetted onto a dispersible and dissolvable porous microcarrier tablet (3D Table Trix, 20 mg/tablet) to allow direct absorption of the suspension hydration of the porous structures. The SSC microcryogels were then maintained in a CO_2_ incubator at 37 °C for 1 h to allow for further cell attachment. Then, the circular bone defect was filled with the SSC microcryogels. The same concentration of microcryogel without SSCs was used as a control.

### Enzyme-linked immunosorbent assay (ELISA)

The concentrations of mouse cytokines from serum samples were measured with TRAP ELISA Kits from CZKWbio (http://www.czkwbio.com/) according to the manufacturer’s protocols.

### Quantitative real-time PCR analysis

Total RNA was extracted using TRIzol reagent (Fermentas) and reverse transcribed using the mRNA Selective PCR Kit (TaKaRa) according to the manufacturer’s instructions. Real-time PCR was performed using a SYBR PCR Master Mix kit (Sigma-Aldrich) and a 7500 Real-Time PCR detection system (Applied Biosystems, ABI). All data were normalized to the control using GAPDH as an internal control. The primers were synthesized by Invitrogen and are listed in Additional file 5: Supplementary Table 1.

### Western blotting analysis

Total proteins were extracted using protein lysis buffer (BioRad, Hercules, CA) containing protease inhibitor cocktail (MCE). A protein extraction kit (Thermo) was used to extract nuclear and cytoplasmic proteins. The concentrations were determined using a BCA kit (Thermo). Equal amounts of protein from each sample were run on a 10% Tris–glycine SDS–PAGE gel, followed by transfer to PVDF membranes (Millipore). Membranes were blocked with 5% skim milk and probed with the indicated primary antibodies overnight at 4 °C. The membranes were washed 3 times with a mixture of Tris-buffered saline and Tween-20, followed by incubation with horseradish peroxidase-conjugated anti-IgG (Cell Signaling) for 1 h at room temperature. The intensities of the bands were visualized and determined using a Tanon-5200 automatic chemiluminescence imaging system (YPH-Bio, Beijing). The primary antibodies used were Akt, p-Akt, GAPDH (1:2000, Cell Signaling) and NRF2, KEAP1, GSK-3β, p-GSK-3β, lamin B1, GCLC, GCLM, HMOX1, and NQO1 (1:1000, ProteinTech). Quantification of the gray values of the bands was performed with ImageJ software.

### μ-computerized tomography (μCT) and analysis

The femurs were fixed in 4% polyoxymethylene for 2 days and then stored in 70% ethanol at 4 °C before being processed. μCT scanning and analysis were performed using a Scanco μCT-40 (Scanco Medical) to evaluate trabecular bone and bone regeneration. The femurs were scanned at a resolution of 8 μm (55 kV, 114 mA, 500 ms integration time), and reconstruction of three-dimensional (3D) images was performed using a standard convolution back-projection. The analyzed data and the quantified parameters, including bone mineral density (BMD), bone volume per tissue volume (BV/TV), trabecular bone thickness (Tb.Th), trabecular bone number (Tb.N), and trabecular separation (Tb.Sp), were determined.

### Calcein labeling assay

C57/BL6N mice were intraperitoneally injected with 20 mg/Kg calcein (Sigma-Aldrich, USA) dissolved in PBS at a concentration of 2 mg/ml with 1 mg/ml NaHCO3 (Sigma-Aldrich, USA) at 7 d and 2 d before killing. After killing, the femurs were obtained, fixed in 4% formaldehyde, and embedded in methyl methacrylate. A hard tissue slicing machine (SP1600; Leica, Germany) was used to sagittally section specimens into 30-mm sections away from light. Then, the cortical endosteum surfaces were evaluated using a fluorescence microscope (STP6000; Leica, Germany) with an excitation wavelength of 488 nm. The bone formation rate (BFR), mineral deposition rate (MAR), and recommended relevant calculations were used for quantitative analysis using ImageJ software.

### Histology and immunohistochemistry

The femurs were decalcified with 10% EDTA and then embedded in paraffin. Five-micrometer coronal sections of the metaphysis and five-micrometer sagittal sections of the bone defect area were prepared, stained with TRAP, hematoxylin–eosin (HE), and Masson, and observed by light microscopy (Zeiss, Germany). For COL-I and OCN immunohistochemical staining, after quenching with endogenous peroxidase, antigen retrieval, and blocking nonspecific binding sites, the femur sections were incubated with anti-COL-I (1:100; Servicebio, China) and anti-OCN (1:100; Servicebio, China).

### Immunofluorescence staining

For immunofluorescence double staining, freshly dissected bone tissues were fixed in 4% paraformaldehyde for 2 days and decalcified in 10% EDTA for 1 weeks. Slides were incubated with anti-CD105 (1:500, Abcam, ab107595), anti-CD200 (1:100, ProteinTech, 14057-1-AP), anti-Rabbit HRP (1:1000, Servicebio, GB23303), and anti-Rabbit 488 (1:1000, Servicebio, GB25303). Images were acquired with fluorescence inverted microscope (Leica, DMIL) and analyzed by ImageJ software.

### Statistical analysis

All data are presented as the mean values with standard deviations. Statistical significance was determined using Student’s t test and one-way analysis. Asterisks denote statistical significance (*P < 0.05; **P < 0.01).

## Results

### Psoralen mitigated irradiation-induced osteoporosis

**Fig. 1:**
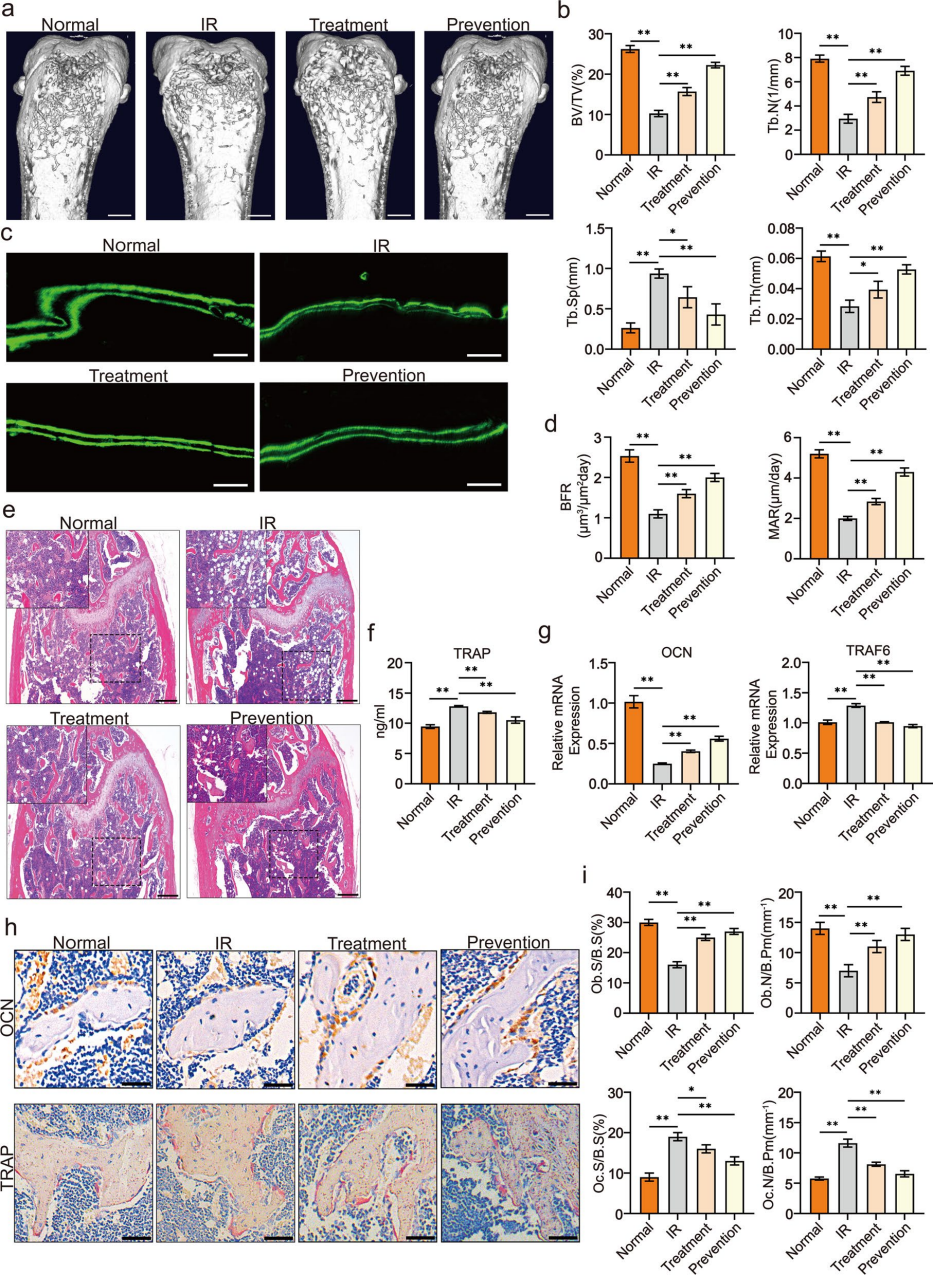
Psoralen mitigated irradiation-induced osteoporosis in a murine model. For the prevention/treatment group, C57BL/6 N mice (n = 5 per group) were administered psoralen (20 mg/Kg) intragastrically every day for 1 week before/after radiation and killed 1 week after radiation. The representative MicroCT data of femur bones at week 1 post-irradiation are shown in **a** and **b**. The results of BV/TV, Tb.Sp, Tb.Th, and Tb.N demonstrated that irradiation induced significant destruction of the bone structures, while treatment or prevention with psoralen remarkably alleviated the bone injuries (**a** and **b**). In addition, the image of calcein double-labeling analysis and quantitative data of BFR and MAR showed that gastric administration of psoralen promoted new bone formation in irradiated mice (**c** and **d**). The HE staining data further demonstrated that psoralen treatment provided protective effects on the bone structures of irradiated mice (**e**). The ELISA data showed that psoralen treatment reduces the TRAP level in serum of irradiated mice (**f**). Gene expression analysis of TRAF6 and OCN in femurs also suggested that psoralen inhibited osteoclastogenesis while favoring osteogenesis in irradiated mice (**g**). Further pathological analysis showed that psoralen partially restored the irradiation induced the reduction of OCN-labeled OBs and the increase of TRAP-labeled OCs in the irradiated mice (**h** and **i**). All data are shown as the mean ± SD. **P < 0.01, *P < 0.05. The scale bars represent 2 mm (**a** and **c**), 500 μm (**e**), and 200 μm (**h**), respectively. IR: irradiation; BV/TV: bone volume per tissue volume; Tb.N: trabecular bone number; Tb.Sp: trabecular separation; Tb.Th: trabecular bone thickness; BFR: bone formation rate; MAR: mineral deposition rate; TRAP: tartrate-resistant acid phosphatase; OCN: osteocalcin; TRAF6: TNF receptor-associated factor 6; Ob.S/B.S: osteoblast surface per bone surface; Ob.N/B.Pm: number of osteoblasts per bone perimeter; Oc.S/B.S: osteoclast surface per bone surface; and Oc.N/B.Pm: number of osteoclasts per bone perimeter

MicroCT images from representative femur bones of each group at week 1 post-irradiation are shown in Fig. 1a. In addition, differences in the cortical and trabecular bone structures of the distal femur metaphysis were analyzed, as shown in Fig. 1b. The microCT images and quantitative data demonstrated that irradiation induced significant degradation of the bone structures, while treatment or prevention with psoralen remarkably alleviated the bone injuries in irradiated mice (Fig. 1a and b). Moreover, calcein double-labeling analysis showed that gastric administration of psoralen before and after irradiation increased new bone formation in irradiated mice (Fig. 1c and d). Consequently, the HE staining data showed that psoralen treatment provided protective effects on the bone structures of irradiated mice, and these results were similar to the microCT results (Fig. 1e). The serum TRAP ELISA data further validated the suppression of irradiation-induced osteoporosis caused by psoralen treatment (Fig. 1f). Gene expression analysis of TRAF6 and OCN in femurs also suggested that psoralen inhibited osteoclastogenesis while favoring osteogenesis in irradiated mice (Fig. 1g). Further pathological staining of osteoblasts (OBs) and osteoclasts (OCs) showed that irradiation led to a notable decrease in OCN-labeled OBs and an increase in TRAP-labeled OCs in the irradiated mice (Fig. 1h and i). Promisingly, in the psoralen treatment and prevention groups, the number of OBs was notably greater than that in the irradiation group (Fig. 1h and i). Conversely, there was a significant decrease in OCs in the presence of psoralen compared with the irradiated mouse group (Fig. 1h and i).

### Psoralen promoted bone regeneration of irradiated bone defects

**Fig. 2:**
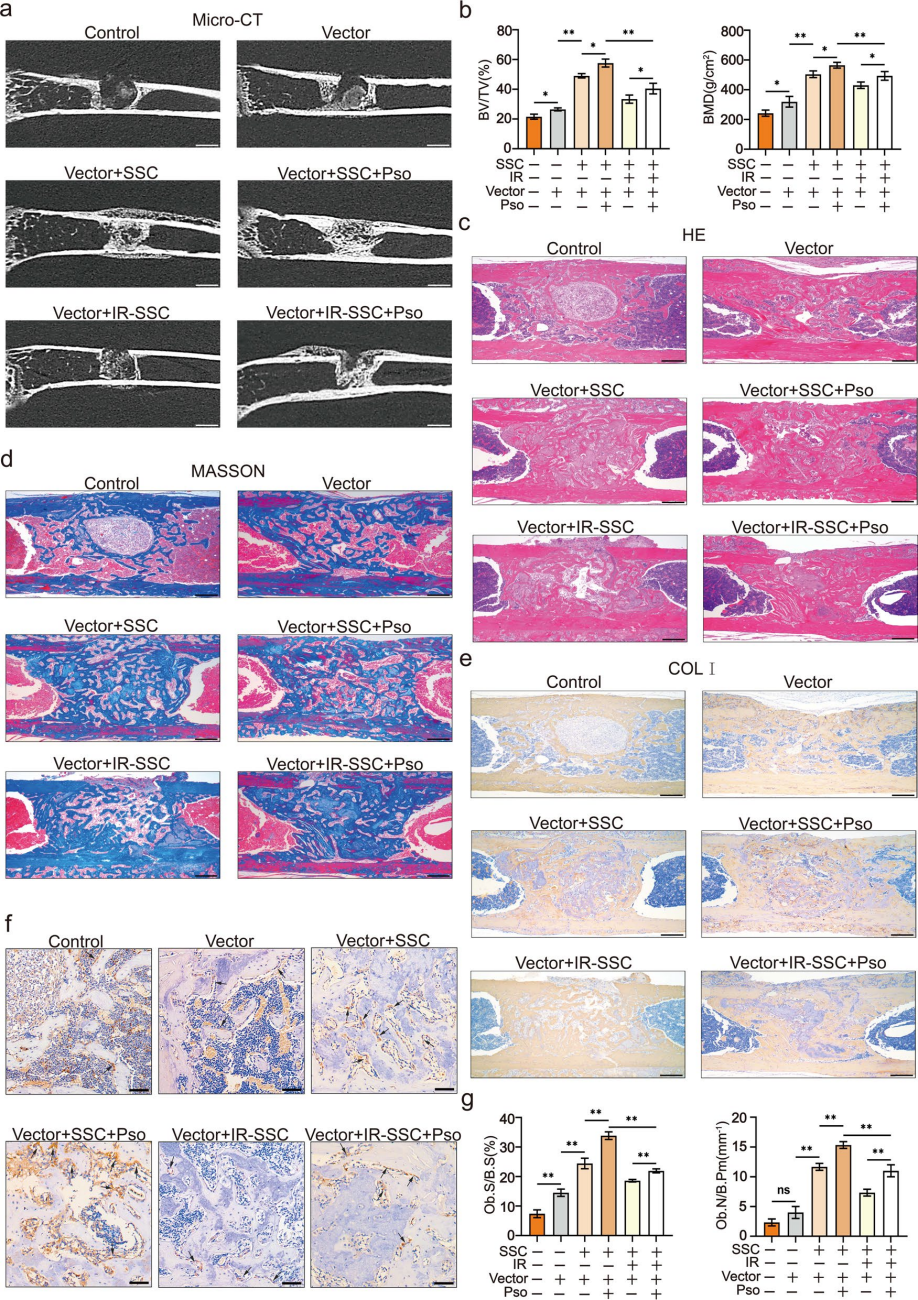
Psoralen promoted bone regeneration of irradiated bone defects in a murine model. The image and quantitative data (BV/TV and BMD) of microCT showed that psoralen pretreatment enhanced the bone regenerative capacity of SSCs in irradiated bone defects (**a** and **b**). In addition, the results of HE, Masson, and Col-I staining demonstrated that Psoralen pretreatment of SSCs or irradiated SSCs yielded better bone formation than their counterparts without psoralen stimulation (**c**–**e**). Moreover, psoralen pretreatment increased OCN-labeled OBs in the irradiated mice transplanted with SSCs or irradiated SSCs (**f** and **g**). All data are shown as the mean ± SD. **P < 0.01, *P < 0.05. The scale bars represent 2 mm (**a**), 500 μm (**c**–**e**), and 200 μm (**f**), respectively. Control: irradiated bone defect; Vector: microgel; SSC: skeletal stem cell; IR: irradiation; Pso: psoralen; BV/TV: bone volume per tissue volume; BMD: bone mineral density; COL-I: collagen I; Ob.S/B.S: osteoblast surface per bone surface; and Ob.N/B.Pm: number of osteoblasts per bone perimeter;

The effects of psoralen on bone regeneration in irradiated bone defects were evaluated by microCT at 2 weeks post-SSC-microcryogel transplantation. As shown in Fig. 2a, more newborn bones were observed in the SSC-microcryogel group than in the negative control group and the microcryogel group. However, there was a decrease in the regenerative capacity of irradiated SSCs with respect to repairing irradiated bone defects. Promisingly, SSCs or irradiated SSCs pretreated with psoralen significantly improved bone formation in irradiated bone defects. MicroCT quantitative analysis showed that BV/TV and BMD were higher in the psoralen-pretreated groups than in groups without psoralen (Fig. 2b). Moreover, HE and Masson staining as well as immunohistochemical analyses of Col-I was performed (Fig. 2c–e, Additional file 1: Fig. S1 and Additional file 2: Fig. S2). The histopathological results demonstrated that SSCs combined with microcryogels developed bone-like tissues that filled most of the defects. However, only small bone nodules were observed in the bone defects that were repaired with irradiated SSCs with the microcryogel. Notably, Psoralen pretreatment of SSCs or irradiated SSCs yielded better bone formation than their counterparts without psoralen stimulation. Notably, the promotive effects of psoralen on bone repair increased in a time-dependent manner (Fig. 2c–e, Additional file 1: Fig. S1 and Additional file 2: Fig. S2). Furthermore, psoralen pretreatment increased OCN-labeled OBs in the irradiated mice transplanted with SSCs or irradiated SSCs (Fig. 2f and g).

### Psoralen rescued the stemness of irradiated SSCs

**Fig. 3:**
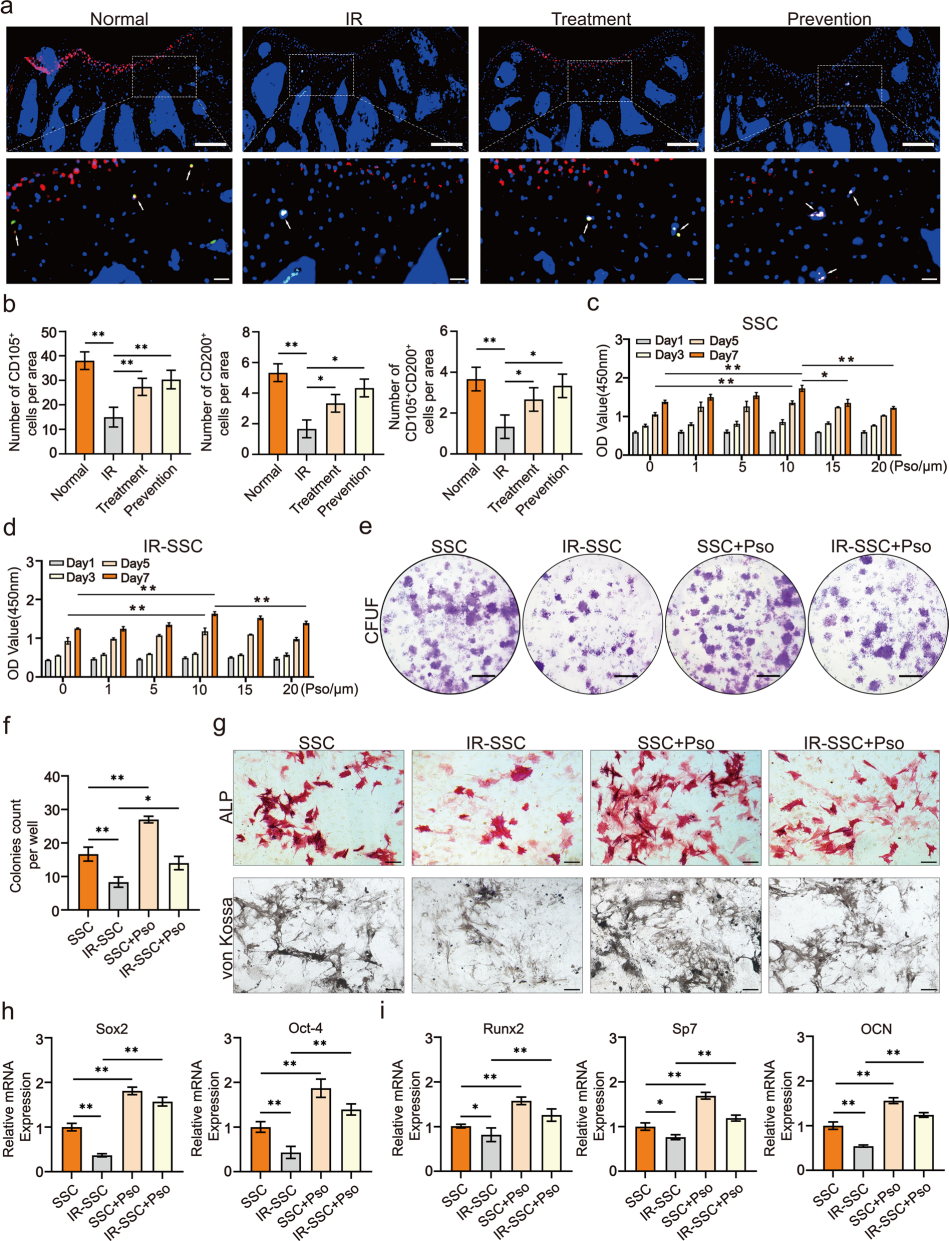
Psoralen rescued the stemness of irradiated SSCs. In **a** and **b**, the data of immunofluorescence staining showed that psoralen partially restored radiation-induced reduction of in situ CD105^+^, CD200^+^, and CD105^+^CD200^+^ cells. The CCK-8 data showed that that psoralen enhanced the proliferation of SSCs and irradiated SSCs in a dose-dependent manner. (**c** and **d**). The CFU-F assay data showed that psoralen significantly promoted colony formation in both the SSC and irradiated SSC groups (Additional file 3: Fig. S3a, Fig. 3**e** and **f**). The results of ALP staining and von Kossa staining demonstrated that psoralen partially rescued the impaired osteogenic capacity post-irradiation (Additional file 3: Fig. S3b, Fig. 3**g**). The expression of self-renewal-related genes, including Sox2 and Oct-4, as well as osteogenesis-related genes, including Runx2, Sp7 and OCN, in irradiated SSCs was partially restored with the addition of psoralen (Additional file 3: Fig. S3c and d, Fig. 3**h** and **i**). All data are shown as the mean ± SD. **P < 0.01, *P < 0.05. The scale bars represent 2 mm (**a**), 5 mm (**e**), and 200 μm (**g**), respectively. CFU-F: colony-forming unit-fibroblast; SSC: skeletal stem cell; IR: irradiation; and Pso: psoralen

Accumulating studies have demonstrated that CD105 and CD200 are pivotal cell surface markers of tissue-specific stem/progenitor cells the skeleton. As shown in Fig. 3a and b, irradiation resulted in a significant reduction in in situ CD105^+^, CD200^+^, and CD105^+^CD200^+^ cells in murine femur bones, while psoralen restored the number of CD105^+^, CD200^+^, and CD105^+^CD200^+^ cells. To investigate the direct effects of psoralen on the regenerative capacity of SSCs, the cell proliferation, colony formation, and osteogenic differentiation of SSCs in the presence of psoralen were evaluated. The results of the CCK-8 assay showed that psoralen enhanced the proliferation of SSCs and irradiated SSCs in a dose-dependent manner, and the promoting effects on SSCs reached their peak at 10 μm psoralen (Fig. 3c and d). In addition, the CFU-F assay data showed that psoralen significantly promoted colony formation in both the SSC and irradiated SSC groups (Additional file 3: Fig. S3a, Fig. 3e and f). Moreover, the results of ALP staining and von Kossa staining suggested that psoralen restored the impaired osteogenic capacity post-irradiation (Additional file 3: Fig. S3b, Fig. 3g). Consistent with the cytochemical staining, the expression of self-renewal-related genes, including Sox2 and Oct-4, as well as osteogenesis-related genes, including Runx2, Sp7 and OCN, in irradiated SSCs was partially restored with the addition of psoralen (Additional file 3: Fig. S3c and d, Fig. 3h and i).

### Psoralen restored irradiation-induced suppression of the PI3K/AKT pathway in SSCs

**Fig. 4:**
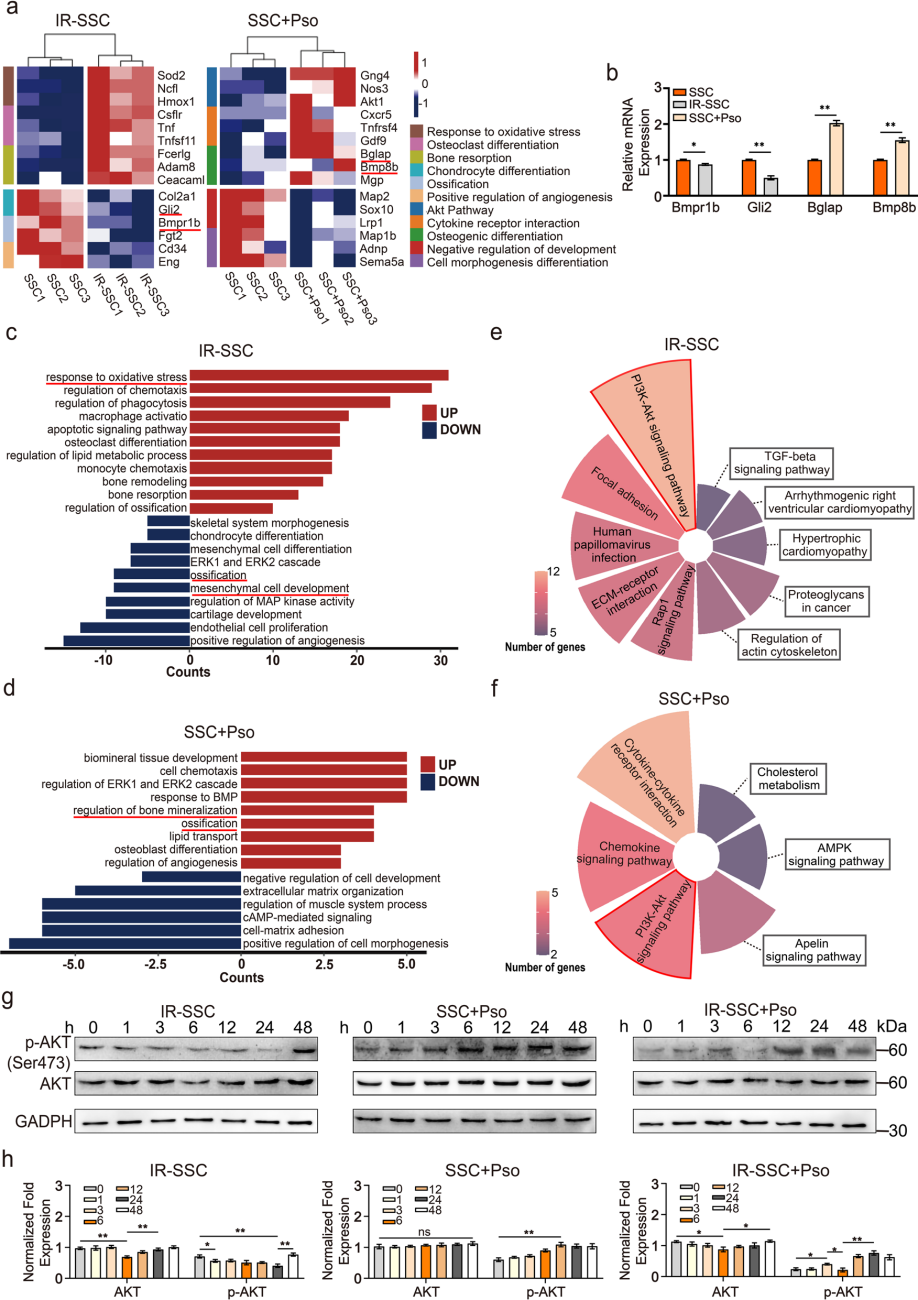
Psoralen restored irradiation-induced suppression of the PI3K/AKT pathway in SSCs. The RNA sequencing data showed that the expression of the osteogenesis-related gene Bmpr1b and the cell proliferation-related gene Gli2 was significantly downregulated in the irradiated SSCs (**a**). In the psoralen-treated SSCs, the expression of the osteogenesis-related genes Bglap and Bmp8b was remarkably upregulated (**a**). The gene expression results of RNA sequencing were validated by qPCR (**b**). The Gene Ontology (GO) analysis results of the irradiation induced the change of gene expression including upregulation of genes responding to oxidative stress and the downregulation of genes related to ossification and mesenchymal cell development, etc., (**c**) while the psoralen induced the upregulation of genes involved in bone mineralization, ossification, etc. (**d**). Further KEGG analysis suggested that the PI3K/AKT signaling pathway was closely involved in both irradiation- and psoralen-induced changes in SSC properties (**e** and **f**). The results of Western Blotting showed that irradiation significantly inhibited the expression and phosphorylation of AKT in SSCs as well as psoralen promoted the activation of AKT in SSCs while increasing AKT expression and phosphorylation in irradiated SSCs (**g** and **h**). All data are shown as the mean ± SD. **P < 0.01, *P < 0.05. SSC: skeletal stem cell; IR: irradiation; and Pso: psoralen

To explore the underlying mechanisms by which psoralen restores the impaired stemness of irradiated SSCs, the mRNA expression profile of SSCs after irradiation and SSCs after psoralen treatment was identified by transcriptome sequencing. The data in Fig. 4a present a heatmap of the differentially expressed genes between SSCs and irradiated SSCs as well as SSCs and psoralen-treated SSCs. Notably, in the irradiated SSCs, the expression of the osteogenesis-related gene Bmpr1b and the cell proliferation-related gene Gli2 was significantly downregulated (Fig. 4a). In the psoralen-treated SSCs, the expression of the osteogenesis-related genes Bglap and Bmp8b was remarkably upregulated (Fig. 4a). The qPCR results validated the gene expression results of RNA sequencing (Fig. 4b). The Gene Ontology (GO) analysis results of the irradiated SSCs showed that all of the differentially abundant genes were mainly involved in the upregulation of genes responding to oxidative stress and the downregulation of genes related to ossification and mesenchymal cell development, etc. (Fig. 4c). The Gene Ontology (GO) analysis results of the psoralen-treated SSCs showed that all of the differentially abundant genes were mainly involved in the upregulation of genes involved in bone mineralization, ossification, etc. (Fig. 4d). Further KEGG analysis suggested that the PI3K/AKT signaling pathway was closely involved in both irradiation- and psoralen-induced changes in SSC properties (Fig. 4e and f). The results of Western Blotting validated the KEGG data from RNA sequencing. Irradiation significantly inhibited the expression and phosphorylation of AKT in SSCs. Notably, psoralen promoted the activation of AKT in SSCs while increasing AKT expression and phosphorylation in irradiated SSCs (Fig. 4g and h).

### Psoralen promoted NRF2 expression and nuclear translocation and increased the production of antioxidants in SSCs

**Fig. 5:**
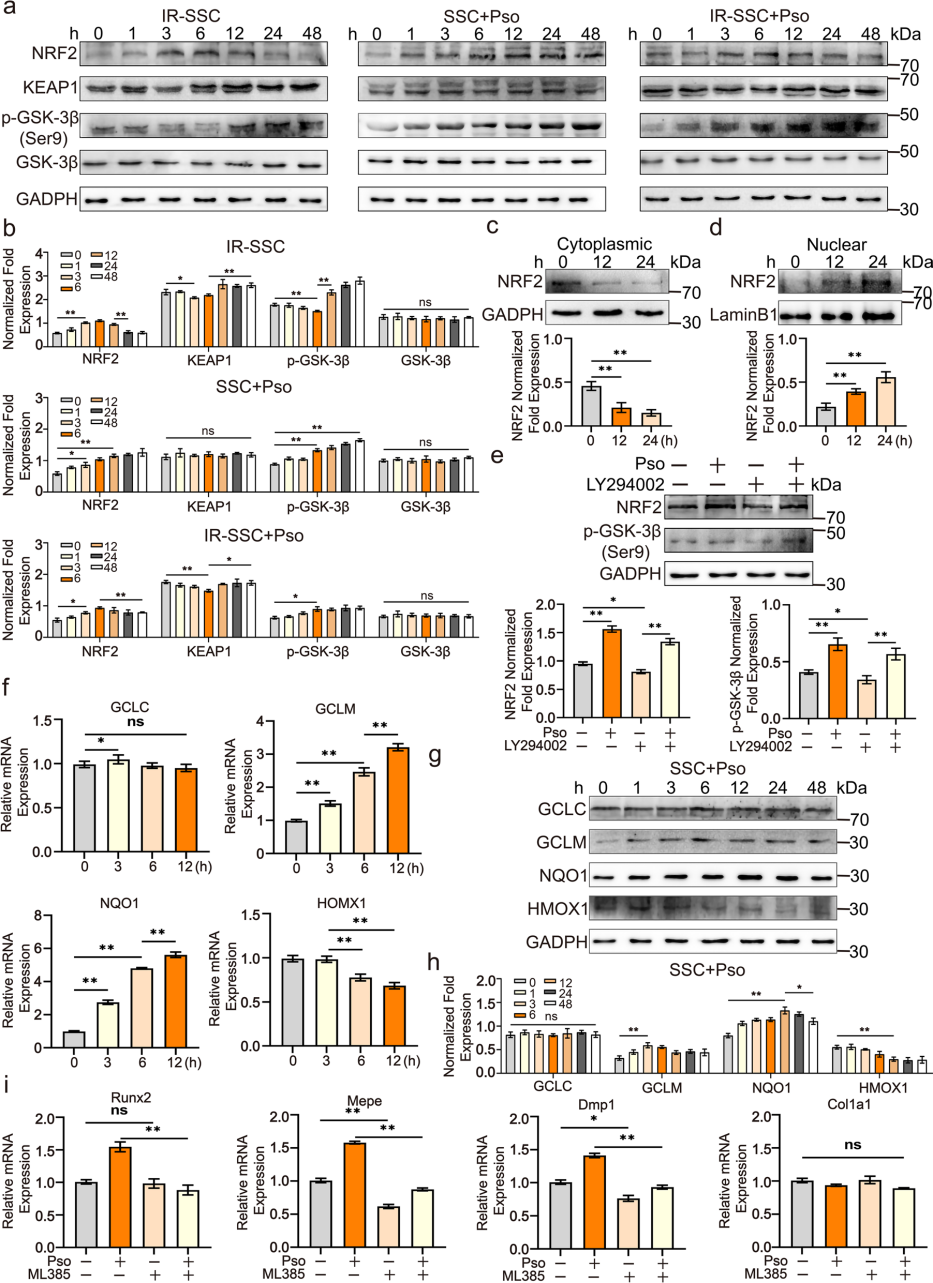
Psoralen promoted NRF2 expression and nuclear translocation and increased the production of antioxidants in SSCs by activating the AKT, GSK-3β, and NRF2. The Western Blotting data showed that the expression of NRF2 in SSCs significantly increased at 3 h and reached a peak at 6 h post-irradiation (**a** and **b**). Psoralen remarkably stimulated the expression of NRF2 in SSCs at 1 h, and these effects lasted for at least 48 h. Notably, psoralen also increased NRF2 expression in irradiated SSCs (**a** and **b**). In addition, irradiation suppressed the expression of KEAP1 in SSCs (**a** and **b**). No significant changes in KEAP1 in SSCs after psoralen treatment were observed. The addition of psoralen to irradiated SSCs strongly increased the NRF2 level but did not change the decrease in KEAP1 (**a** and **b**). Moreover, the Western Blotting data showed that irradiation significantly suppressed the phosphorylation of GSK-3β, while psoralen promoted the phosphorylation of GSK-3β in SSCs (**a** and **b**). Furthermore, the Western Blotting data showed that psoralen significantly decreased cytoplasmic NRF2 but increased nuclear NRF2 in a time-dependent manner (**c** and **d**). A specific chemical inhibitor of AKT, LY294002 (5 μM), significantly decreased GSK-3β phosphorylation and NRF2 expression in the presence or absence of psoralen (**e**). The mRNA expression and protein levels of GCLM and NQO1 were significantly induced, while that of HOMX1 was inhibited by psoralen in SSCs (**f**–**h**). ML385, a specific chemical inhibitor of NRF2, significantly suppressed the expression of osteogenic genes, including Runx2, Mepe, and Dmp1, in SSCs in the presence of psoralen (**i**). All data are shown as the mean ± SD. **P < 0.01, *P < 0.05. SSC: skeletal stem cell; IR: irradiation; and Pso: psoralen

Because NRF2 is one of the pivotal transcription factors in the defense against oxidative stress, the expression of NRF2 in irradiated SSCs was detected by Western Blotting. The data showed that the expression of NRF2 in SSCs significantly increased at 3 h and reached a peak at 6 h post-irradiation (Fig. 5a and b). Promisingly, psoralen remarkably stimulated the expression of NRF2 in SSCs at 1 h, and these effects lasted for at least 48 h. Notably, psoralen also increased NRF2 expression in irradiated SSCs (Fig. 5a and b). KEAP1 has been identified as a key cofactor of NRF2-mediated regulation by promoting NRF2 degradation. Thus, KEAP1 expression in SSCs post-irradiation/psoralen treatment was detected. Consistent with previous reports, irradiation suppressed the expression of KEAP1 in SSCs, which suggests that the elevation of NRF2 may occur by targeting KEAP1 in irradiated SSCs (Fig. 5a and b). However, no significant changes in KEAP1 in SSCs after psoralen treatment were observed in the current study, which indicated that novel underlying mechanisms increased the expression of NRF2 in SSCs. Moreover, the addition of psoralen to irradiated SSCs strongly increased the NRF2 level but did not change the decrease in KEAP1, which suggested that irradiation and psoralen induced NRF2 expression via different pathways (Fig. 5a and b). Because GSK-3β promotes NRF2 degradation, while phosphorylated GSK-3β turns to lose their capacity to degrade NRF2, therefore, the expression and phosphorylation of GSK-3β in SSCs in response to irradiation and psoralen are also shown in Fig. 5a and b. The Western Blotting data showed that irradiation significantly suppressed the phosphorylation of GSK-3β, while psoralen promoted the phosphorylation of GSK-3β in SSCs.

In addition to the effects of psoralen on the expression of NRF2 in SSCs, the possibility of NRF2 nuclear translocation after psoralen treatment was also investigated in the current study. The Western Blotting data showed that psoralen significantly decreased cytoplasmic NRF2 but increased nuclear NRF2 in a time-dependent manner (Fig. 5c and d).

To further explore the upstream signaling pathway that controls NRF2 levels in SSCs and based on the results that PI3K/AKT significantly changed after both irradiation and psoralen treatment, a specific chemical inhibitor of AKT, LY294002, was added to the SSC culture system. The appropriate concentration of LY294002 was screened by using Western Blotting and CCK-8 assays (Additional file 4: Fig. S4a and b). LY294002 (5 μM) significantly decreased GSK-3β phosphorylation and NRF2 expression in the presence or absence of psoralen, which suggested that AKT controls GSK-3β and NRF2 in SSCs and may contribute to psoralen-mediated NRF2 elevation (Fig. 5e).

GCLC, GCLM, NQO1, and HOMX1 are well-known downstream genes of NRF2 that are involved in antioxidant production. In the current study, the mRNA expression levels of GCLM and NQO1 were significantly induced, while that of HOMX1 was inhibited by psoralen at different time points. Consistent with the mRNA data, the Western Blotting results showed that psoralen increased the expression of GCLM and NQO1 but inhibited the expression of HOMX1 in SSCs (Fig. 5f–h). In addition, NRF2 is closely involved in osteogenesis. Thus, ML385, a specific chemical inhibitor of NRF2, was used to block the potential effects of psoralen-induced osteogenic differentiation via NRF2. The appropriate concentration of ML385 was screened by using Western Blotting and CCK-8 assays (Additional file 4: Fig. S4c and d). Blocking NRF2 with the specific chemical inhibitor ML385 significantly suppressed the expression of osteogenic genes, including Runx2, Mepe, and Dmp1, in SSCs in the presence of psoralen (Fig. 5i).

### Blockade of PI3K/AKT and NRF2 partially abolished the promotion of psoralen-induced the proliferation and osteogenic activity of SSCs

**Fig. 6:**
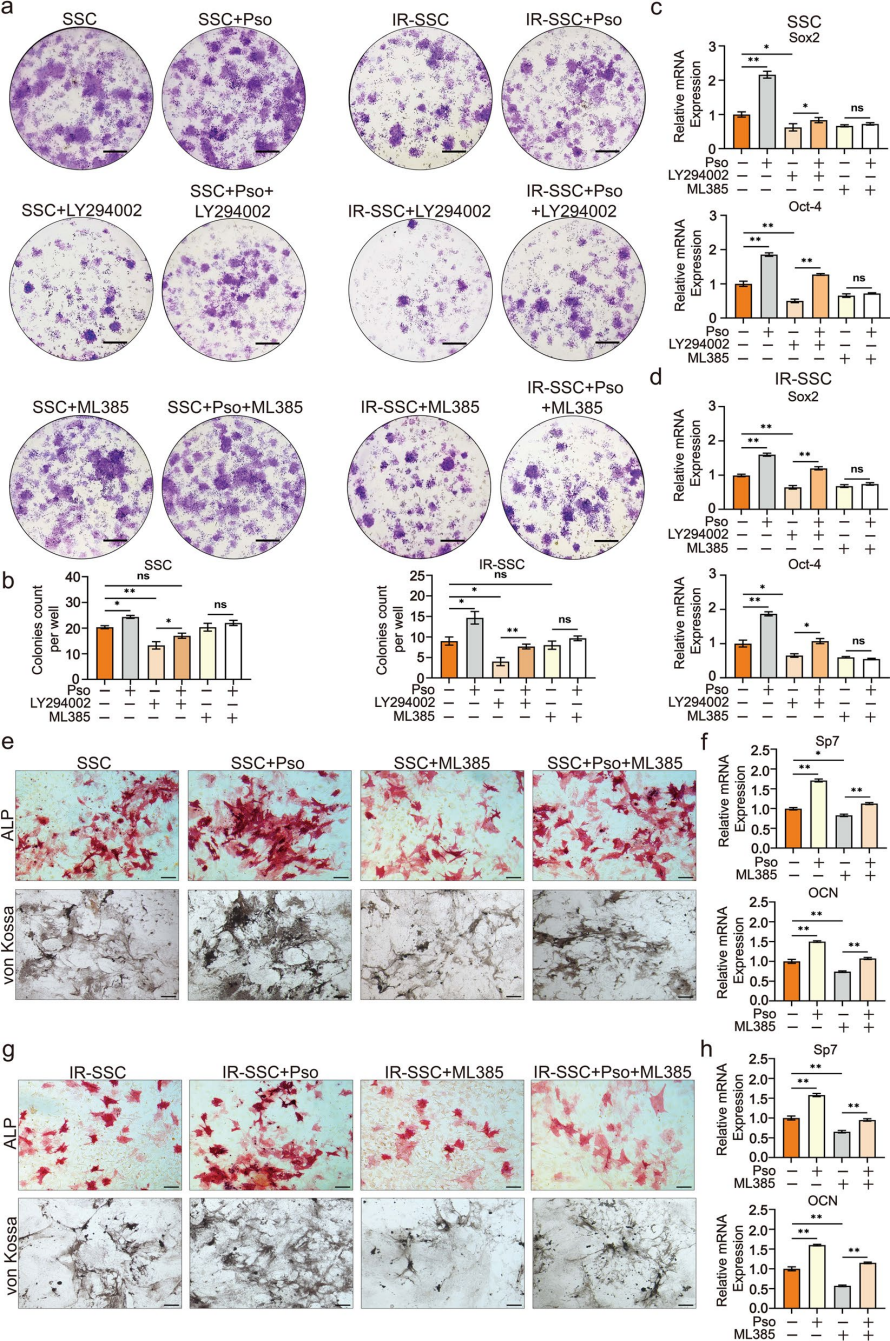
Blockade of PI3K/AKT and NRF2 partially abolished the promotion of psoralen-induced the proliferation and osteogenic activity of SSCs. As shown in **a** and **b**, blockage of AKT by LY294002 significantly abolished the psoralen-induced colony formation in SSCs and irradiated SSCs. The changes in the mRNA levels of the self-renewal-related genes Sox2 and Oct-4 further validated the CFU-F data (**c** and **d**). The ALP and von Kossa staining results showed that supplementation with ML385 (10 μM) partially abolished psoralen-induced osteogenesis in SSCs and irradiated SSCs (**e** and **g**). qPCR data showed that the expression of the osteogenic genes Sp7 and OCN was suppressed in the presence of ML385 (**f** ad **h**). All data are shown as the mean ± SD. **P < 0.01, *P < 0.05. The scale bars represent 5 mm (**a**) and 200 μm (**e** and **g**), respectively. SSC: skeletal stem cell; IR: irradiation; and Pso: psoralen

Although molecular data have suggested that PI3K/AKT and NRF2 play a role in the SSC response to radiation and psoralen, the stemness of SSCs needs to be further elucidated. As shown in Fig. 6a and b, blockage of AKT by LY294002 significantly abolished the psoralen-induced colony formation in SSCs and irradiated SSCs. However, no obvious differences in the colony number of SSCs or irradiated SSCs were observed with the addition of the NRF2 inhibitor ML385. These data suggest that psoralen may maintain SSC self-renewal by activating the AKT pathway instead of NRF2. The changes in the mRNA levels of the self-renewal-related genes SOX4 and OCT-4 further validated the CFU-F data (Fig. 6c and d).

Because ML385 influenced osteogenic genes in SSCs, the osteogenic activity of SSCs in the presence of psoralen and/or ML385 was further analyzed. The ALP and von Kossa staining results showed that supplementation with ML385 (10 μM) partially abolished psoralen-induced osteogenesis in SSCs and irradiated SSCs (Fig. 6e and g). Furthermore, qPCR data showed that the expression of the osteogenic genes Sp7 and OCN was suppressed in the presence of ML385 (Fig. 6f ad h).

### Blockage of NRF2 partially abolished the protective effects of psoralen on radiation-induced bone injury in mice

**Fig. 7:**
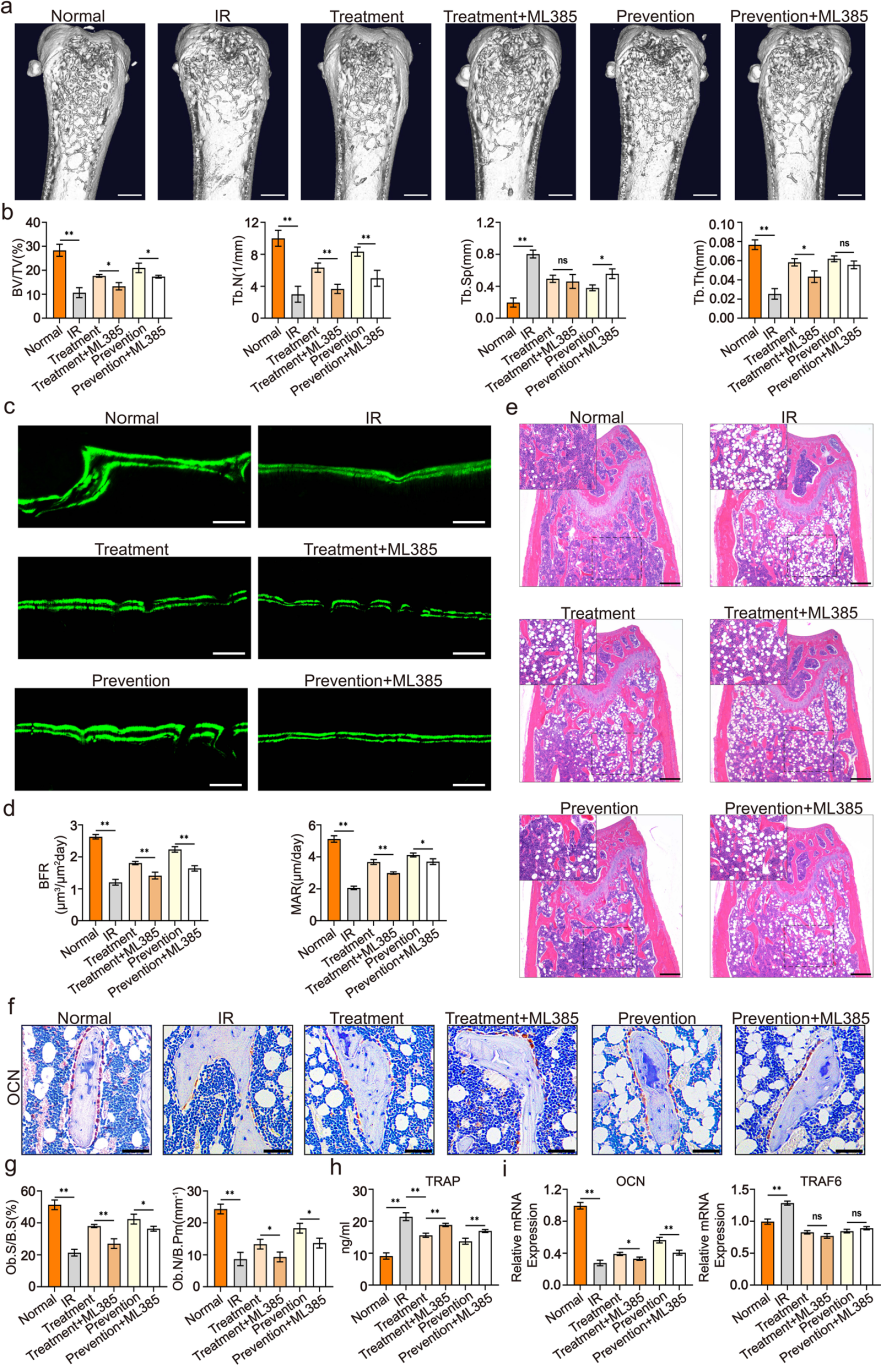
Blockage of NRF2 partially abolished the protective effects of psoralen on radiation-induced osteoporosis in mice. A specific chemical inhibitor of NRF2 (ML385, 30 mg/Kg) was intraperitoneally injected 1 h before psoralen treatment. The microCT images and quantitative data demonstrated that the protective effects of psoralen on bone structure were remarkably impaired in the ML385 administration groups (**a** and **b**). The results of the calcein double-labeling analysis showed that blockade of NRF2 significantly abolished psoralen-mediated new bone formation in irradiated mice (**c** and **d**). The HE staining data showed that ML385 remarkably blunted the rescue and prevented the effects of psoralen on the bone structure of irradiated mice (**e**). OCN staining data showed that ML385 blunted the restorative effects of psoralen on osteoblasts in irradiated mice (**f** and **g**). The serum TRAP ELISA data further validated the blunting of ML385 on the therapeutic effects of psoralen on irradiation-induced osteoporosis (**h**). Gene expression analysis of OCN in femur bones indicated that ML385 led to a turnover of the promotion of bone formation and suppression on bone resorption by psoralen. (**i**). All data are shown as the mean ± SD. **P < 0.01, *P < 0.05. The scale bars represent 2 mm (**a** and **c**), 500 μm (**e**), and 200 μm (**f**), respectively. IR: irradiation; BV/TV: bone volume per tissue volume; Tb.N: trabecular bone number; Tb.Sp: trabecular separation; Tb.Th: trabecular bone thickness; BFR: bone formation rate; MAR: mineral deposition rate; TRAP: tartrate-resistant acid phosphatase; OCN: osteocalcin; TRAF6: TNF receptor-associated factor 6; Ob.S/B.S: osteoblast surface per bone surface; Ob.N/B.Pm: number of osteoblasts per bone perimeter; Oc.S/B.S: osteoclast surface per bone surface; and Oc.N/B.Pm: number of osteoclasts per bone perimeter

To explore the underlying mechanisms that control psoralen-mediated protection against radiation-induced osteoporosis in vivo, a specific chemical inhibitor of NRF2 (ML385, 30 mg/Kg) was intraperitoneally injected 1 h before psoralen treatment. The microCT images and quantitative data demonstrated that the protective effects of psoralen on bone structure were remarkably impaired in the ML385 administration groups (Fig. 7a and b). In addition, the results of the calcein double-labeling analysis showed that blockade of NRF2 significantly abolished psoralen-mediated new bone formation in irradiated mice (Fig. 7c and d). Moreover, the HE staining data showed that ML385 remarkably blunted the rescue and prevented the effects of psoralen on the bone structure of irradiated mice (Fig. 7e). Furthermore, OCN staining data showed that ML385 blunted the restorative effects of psoralen on osteoblasts in irradiated mice (Fig. 7f and g). The serum TRAP ELISA data further validated the blunting of ML385 on the therapeutic effects of psoralen on irradiation-induced osteoporosis (Fig. 7h). Gene expression analysis of OCN in femur bones indicated that ML385 led to a turnover of the promotion of bone formation and suppression on bone resorption by psoralen (Fig. 7i).

**Fig. 8:**
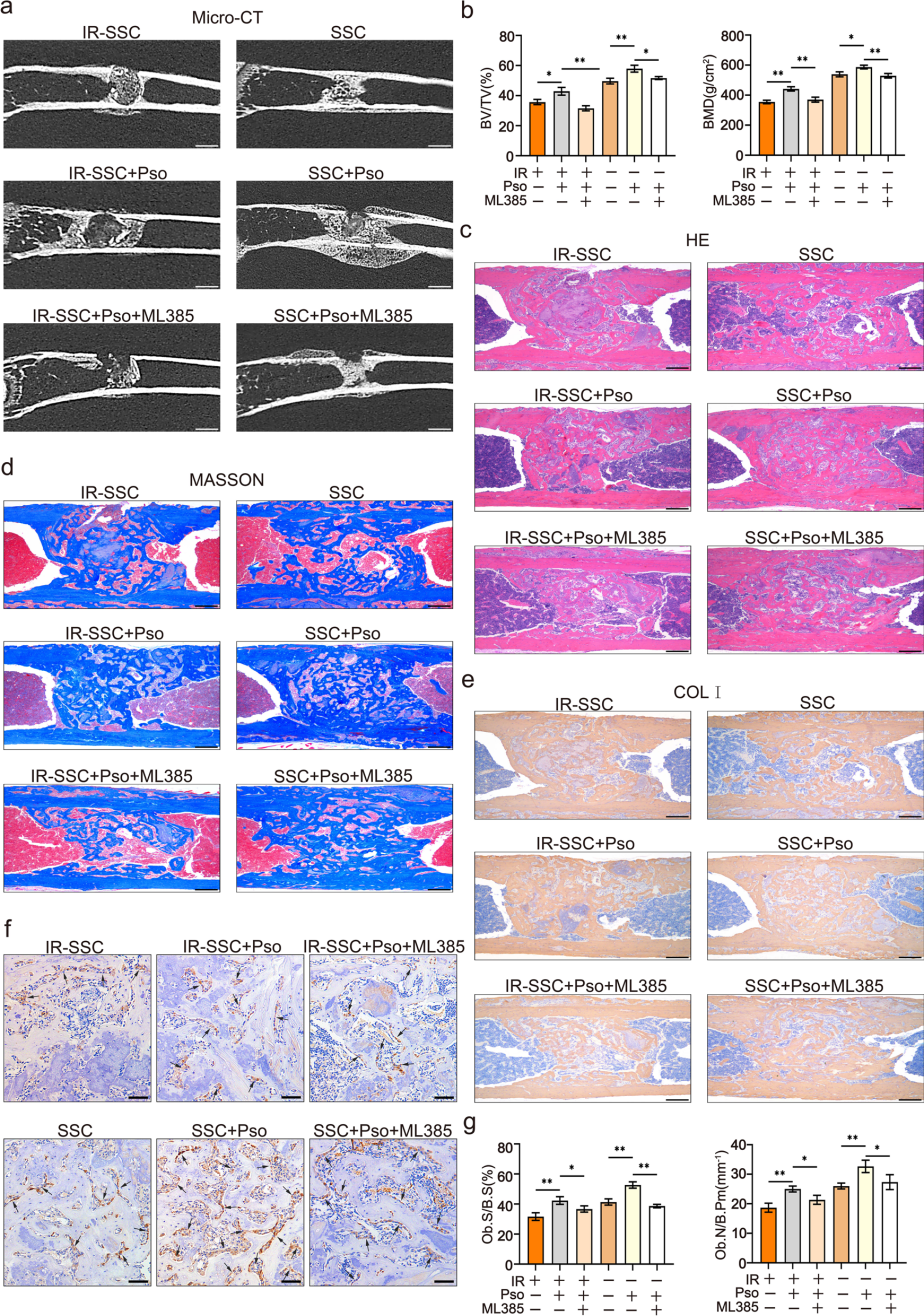
Blockage of NRF2 partially abolished the regenerative effects of psoralen on irradiated bone defects in mice. The microCT results showed that the volume of newborn bones in both the groups of SSCs treated with psoralen and the irradiated SSCs psoralen was lower in the presence of ML385 than in the absence of ML385 (**a** and **b**). The results of HE, Masson, and Col-I staining showed that ML385 administration partially abolished the ability of psoralen to promote the rebuilding of bone structure and collagen formation in the new bones of bone defects caused by irradiation (**c**–**e**). The number of OCN-labeled OBs decreased in the presence of ML385 compared to their counterparts—without NRF2 blockade (**f** and **g**). All data are shown as the mean ± SD. **P < 0.01, *P < 0.05. The scale bars represent 2 mm (**a**), 500 μm (2**c**–**e**), and 200 μm (2f), respectively. Control: irradiated bone defect; Vector: microgel; SSC: skeletal stem cell; IR: irradiation; Pso: psoralen; BV/TV: bone volume per tissue volume; BMD: bone mineral density; Ob.S/B.S: osteoblast surface per bone surface; and Ob.N/B.Pm: number of osteoblasts per bone perimeter

The role of NRF2 in psoralen-mediated bone regeneration in irradiated bone defects was also investigated in vivo. The microCT results showed that the volume of newborn bones in both the groups of SSCs treated with psoralen and the irradiated SSCs psoralen was lower in the presence of ML385 than in the absence of ML385 (Fig. 8a and b). Additionally, the results of HE, Masson, and Col-I staining showed that ML385 administration abolished the ability of psoralen to promote the rebuilding of bone structure and collagen formation in the new bones of bone defects caused by irradiation (Fig. 8c–e). Furthermore, the number of OCN-labeled OBs decreased in the presence of ML385 compared to their counterparts without NRF2 blockade (Fig. 8f and g).

## Discussion

In the present study, we report that psoralen is capable of alleviating radiation-induced bone injury by rescuing the stemness of SSCs. Mechanistically, we found that activation of the AKT, GSK-3β, and NRF2 partially contributed to the therapeutic effects of psoralen on radiation-induced bone injury. Our findings revealed a novel role of psoralen in the prevention and treatment of irradiation-induced skeletal injury and identified skeletal tissue-specific stem cells as potential cellular targets for antiradiation drugs.

In the current study, female C57BL/6 N mice were exposed to gamma-radiation at 2 Gy doses representing a clinical treatment fraction of radiotherapy [31]. Irradiation-induced bone injuries, such as osteoporosis, and retarded tissue regeneration in irradiated bone defects are common and clinically significant [32]. However, limited progress has been made in the prevention and treatment of bone injuries in recent decades, partially due to the incomplete understanding of pathological events and the shortage of bone-specific antiradiation drugs. Unlike other radiation-sensitive tissues, including bone marrow and the intestines, the role of tissue-specific stem cells in the skeleton in response to irradiation and their potential value when seeking drugs have been ignored for a long time. Fortunately, the continued progression in SSC identification and application makes it easier to develop SSC-based anti-irradiation strategies. In past decades, we have pursued the identification of tissue-specific stem cells from skeletal tissues and explored their applications [10, 12, 25, 26, 33–38]. Our previous data demonstrated the pivotal role of SSCs to control the inflammatory response and regulate bone remodeling [26, 36]. Notably, by virtue of the SSC cell model in vitro and the irradiated bone defect model in vivo, we found that irradiation led to an impairment in the regenerative capacity of SSCs and that targeting SSCs by using the antioxidant ferulic acid could partially rescue SSC-mediated tissue regeneration [12]. However, the underlying mechanisms of the SSC response to this drug need to be further explored. In addition, the pathological role of irradiation and its potential value as a cellular drug target need to be further validated in more radiation-induced bone injury models by using more drugs.

In contrast to ferulic acid, psoralen has long been considered a potent component of medicines to treat bone disorders because it promotes bone formation and inhibits bone resorption [16, 17]. However, to the best of our knowledge, less information is available about the role of psoralen on the regulation of antioxidant activities. Notably, in the current study, we unexpectedly found that psoralen strongly increased the expression of NRF2 and promoted antioxidant production in SSCs. First, our data showed that psoralen induced NRF2 expression in an irradiation-independent manner because direct NRF2 supplementation into the SSC culture system significantly resulted in NRF2 upregulation, which suggests that it is possible to prevent bone injury before radiation exposure by preadministration of psoralen. Promisingly, the in vivo radiation osteoporosis models further validated the preventive effects of psoralen. Second, our data show that psoralen induction of NRF2 can last for a relatively long time, which indicates the possibility of the uptake of psoralen to maintain therapeutic effects. Third, psoralen induced NRF2 in a KEAP1-independent manner. The Western Blotting data showed that irradiation induced NRF2 expression and suppressed KEAP1 expression in SSCs. However, psoralen stimulated NRF2 in SSCs without significantly changing the expression of KEAP1. RNA sequencing of SSCs and the Western Blotting data showed that irradiation inactivated AKT, while psoralen promoted the expression and phosphorylation of AKT in irradiated SSCs. Numerous studies have demonstrated that AKT phosphorylation can increase the level of phosphorylated GSK-3β [39–41]. Importantly, it has been reported that phosphorylated GSK-3β remarkably loses its ability to promote NRF2 degradation [42–44]. Consistent with previous reports, we found that psoralen significantly increased the level of phosphorylated GSK-3β in SSCs, which suggested that psoralen may elevate the NRF2 pathway in SSCs in party via the AKT-induced upregulation of GSK-3β and NRF2. Fourth, psoralen significantly increased the level of nuclear NRF2 but decreased the level of cytoplasmic NRF2 in SSCs, which suggests that psoralen may enhance NRF2-mediated antioxidant effects by promoting the translocation of NRF2. Thus, our data suggested that psoralen exhibited modulatory effects on the expression of NRF2 via multiple molecular mechanisms in SSCs. In addition to antioxidant effects, NRF2 has been identified in the past decade as a new player in osteogenesis [45, 46]. However, conflicting data have confused the origin of NRF2 in bone formation, which may lie in the different regulatory effects of NRF2 on specific cell types in osteogenesis [45, 46]. In the current study, blockade of NRF2 by using the specific chemical inhibitor ML385 significantly abolished the psoralen-induced expression of bone formation genes. Moreover, further functional assays in the presence of ML385 confirmed the pivotal role of NRF2 in psoralen-mediated SSC osteogenesis.

Nevertheless, we must acknowledge several limitations in our study. First, high-throughput investigations may be needed in future studies to further identify the subpopulation of SSCs that respond to irradiation and psoralen, which may lead to further understanding of the cellular and molecular mechanisms of the psoralen-mediated therapeutic effects on radiation-induced bone injury. Second, we are aware that although we have identified that the AKT and GSK-3β/NRF2 pathway contributes to the regulatory effects of psoralen on SSCs, the RNA sequencing data suggest that the potential role of other pathways, including the TGF-β/SMAD and AMPK pathways, should be explored in future studies. Third, SSC-specific NRF2-deficient mice will be helpful to exclude the possible influence of other types of bone formation cells on the in vivo therapeutic effects of psoralen.

## Conclusion

In conclusion, the current study revealed for the first time the therapeutic role of psoralen in radiation-induced bone injury, which works to restore the stemness of irradiated SSCs via the AKT-mediated upregulation of GSK-3β and NRF2. These findings indicate that targeting SSCs could be a promising therapeutic strategy to prevent and treat irradiation-induced skeletal disorders.

## Supplementary Information

**Additional file 1. Figure S1**. The promotive effects of Psoralen on the bone regeneration of irradiated bone defects at 1 weeks post SSC transplantation. The results of HE (Fig. S1a), Masson (Fig. S1b) and Col-I (Fig. S1c) staining demonstrated that Psoralen pretreatment of SSCs or irradiated SSCs yielded better bone formation than their counterparts without psoralen stimulation. The Scale bars represent 200 μm (Fig. S1a, S1b an S1c), respectively. Control: irradiated bone defect; Vector: microgel; SSC: skeletal stem cell; IR: irradiation; Pso: psoralen; COL-I: collagen I.

**Additional file 2. Figure S2**. The promotive effects of Psoralen on the bone regeneration of irradiated bone defects at 3 weeks post SSC transplantation. The results of HE (Fig. S2a), Masson (Fig. S2b) and Col-I (Fig. S2c) staining demonstrated that more newborn bones formed in mice of the Psoralen pretreatment of SSCs or irradiated SSC groups. The Scale bars represent 200 μm (Fig. S2a, S2b an S2c), respectively. Control: irradiated bone defect; Vector: microgel; SSC: skeletal stem cell; IR: irradiation; Pso: psoralen; COL-I: collagen I.

**Additional file 3. Figure S3**. Psoralen promote the self-renewal and osteogenic differentiation of SSCs. The results of the CFU-F assay demonstrated that psoralen significantly promoted the cell colony formation of the SSC (Fig. S3a). In addition, the results of ALP staining and von Kossa staining showed that that psoralen promoted osteogenic differentiation of SSCs (Fig. S3b). The expression of self-renewal-related genes, including Sox2 and Oct-4, and osteogenesis-related genes, including Runx2, Sp7 and OCN, in SSCs was significantly upregulated in the presence of psoralen (Fig. S3c and S3d).

**Additional file 4. Figure S4**. The screening of the appropriated concentration of LY294002 and ML385 for SSC proliferation and NRF2 expression in SSCs. The appropriate concentration of LY294002 was screened by using western blotting and CCK-8 assays (Fig. S4a and S4b). The appropriate concentration of ML385 was screened by using western blotting and CCK-8 assays (Fig. S4c and S4d).

**Additional file 5. Supplementary Table 1.** Primers used for RT-PCR analysis.

## Data Availability

The datasets used and/or analyzed during the current study are available from the corresponding author on reasonable request. In addition, the RNA sequencing data for IR-SSC and Psoralen-SSC have been archived and the assigned accession of the submission is: CRA006548 and CRA006500, respectively. Please access it from the following link:https://bigd.big.ac.cn/gsa/browse/CRA006548 for IR-SSC, and https://bigd.big.ac.cn/gsa/browse/CRA006500 for Psoralen-SSC.

## Abbreviations

KEAP1: Kelch-like ECH-associated protein 1
NRF2: Nuclear factor erythroid 2 like 2
SSCs: Skeletal stem cells
IR: Irradiation
α-MEM: Alpha minimum essential medium
FBS: Fetal bovine serum
Pso: Psoralen
OC: Osteoclast
OB: Osteoblast
RNA-Seq: RNA sequencing
microCT: Microcomputed tomography
BMD: Bone mineral density
Tb.Th: Trabecular bone thickness
Tb.N: Trabecular bone number
BV/TV: Bone volume per tissue volume
Tb.Sp: Trabecular separation
BFR: Bone formation rate
MAR: Mineral deposition rate
TRAP: Tartrate-resistant acid phosphatase
ELISA: Enzyme-linked immunosorbent assay
NQO1: NAD(P)H quinone dehydrogenase 1
HMOX1/HO-1: Heme oxygenase 1
GCLM: Glutamate–cysteine ligase modifier
GCLC: Glutamate–cysteine ligase catalytic
TRAF6: TNF receptor-associated factor 6
OCN: Osteocalcin
CFU-F: Colony-forming unit-fibroblast
TRAP: Tartrate-resistant acid phosphatase
