# Correction to: Psoralen alleviates radiation-induced bone injury by rescuing skeletal stem cell stemness through AKT mediated up-regulation of GSK-3β and NRF2

**DOI:** 10.1186/s13287-022-03081-x

**Published:** 2022-07-27

**Authors:** Bo-Feng Yin, Zhi-Ling Li, Zi-Qiao Yan, Zheng Guo, Jia-Wu Liang, Qian Wang, Zhi-Dong Zhao, Pei-Lin Li, Rui-Cong Hao, Meng-Yue Han, Xiao-Tong Li, Ning Mao, Li Ding, Da-Fu Chen, Yue Gao, Heng Zhu

**Affiliations:** 1 Beijing Institute of Radiation Medicine, Road Taiping 27, Beijing, 100850 People’s Republic of China; 2 Beijing Key Laboratory for Radiobiology, Beijing Institute of Radiation Medicine, Beijing, 100850 People’s Republic of China; 3 People’s Liberation Army General Hospital, Road Fuxing 28, Beijing, 100853 People’s Republic of China; 4 Medical Center of Air Forces, PLA, Road Fucheng 30, Beijing, 100142 People’s Republic of China; 5 Graduate School of Anhui Medical University, 81 Meishan Road, Shushan Qu, Hefei, 230032 Anhui People’s Republic of China; 6 Beijing Institute of Basic Medical Sciences, Road Taiping 27, Beijing, 100850 People’s Republic of China; 7 Laboratory of Bone Tissue Engineering, Beijing Laboratory of Biomedical Materials, Beijing Research Institute of Traumatology and Orthopaedics, Beijing Jishuitan Hospital, Eastern Street Xinjiekou 31, Beijing, 100035 China

## Correction to: Stem Cell Research & Therapy (2022) 13:241 10.1186/s13287-022-02911-2

Following publication of the original article [1], the authors have identified that the incorrect image of micro-CT scanning for normal group in Fig. 1a was included due to an error during figure preparation. The corrected image of micro-CT scanning for normal group has been updated in Fig. 1a. Therefore, the revised Fig. 1 is given in this article.

**Fig. 1:**
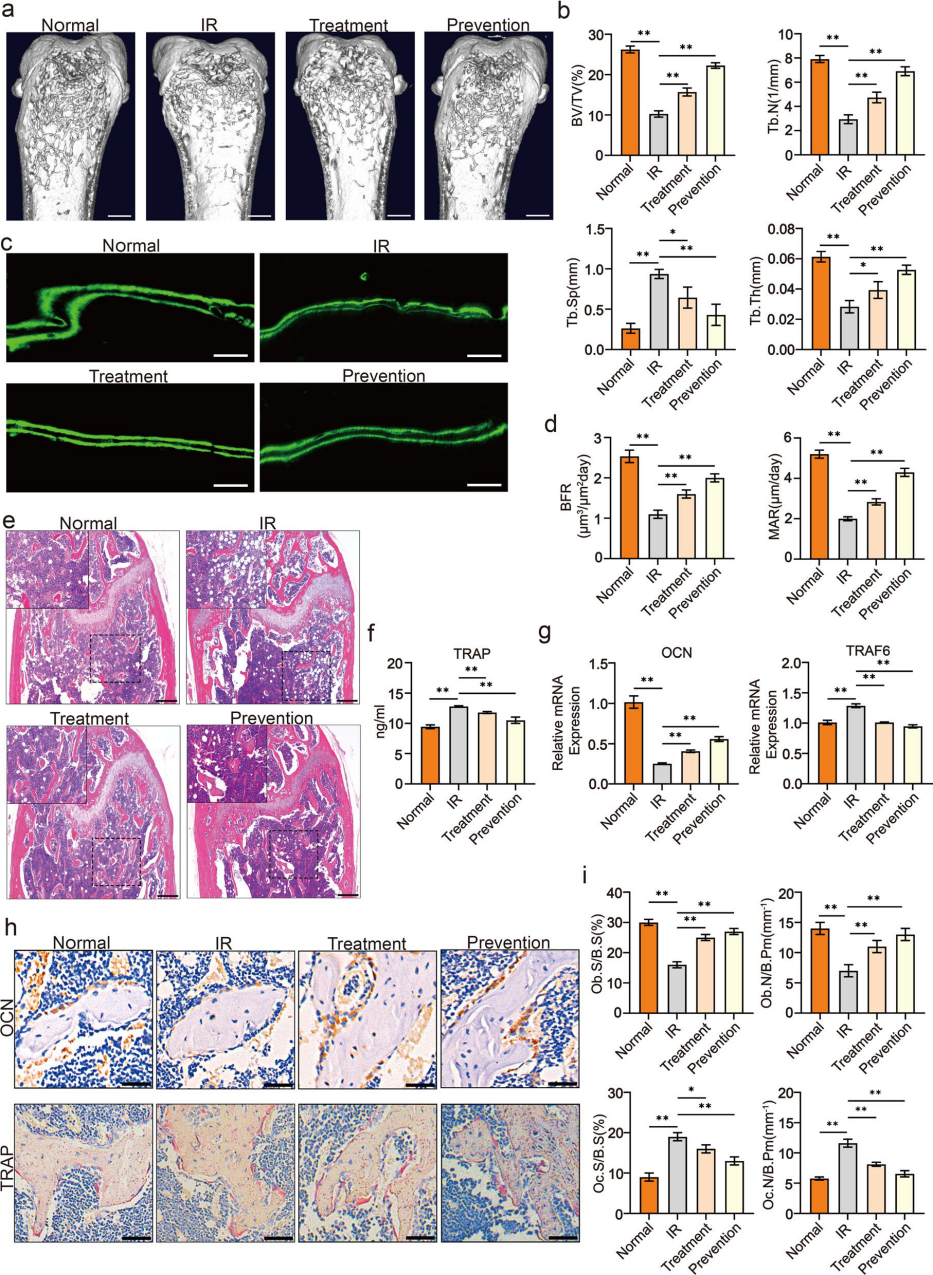
Psoralen mitigated irradiation-induced osteoporosis in a murine model. For the prevention/treatment group, C57BL/6 N mice (n = 5 per group) were administered psoralen (20 mg/Kg) intragastrically every day for 1 week before/after radiation and killed 1 week after radiation. The representative MicroCT data of femur bones at week 1 post-irradiation are shown in **a** and **b**. The results of BV/TV, Tb.Sp, Tb.Th, and Tb.N demonstrated that irradiation induced significant destruction of the bone structures, while treatment or prevention with psoralen remarkably alleviated the bone injuries (**a** and **b**). In addition, the image of calcein double-labeling analysis and quantitative data of BFR and MAR showed that gastric administration of psoralen promoted new bone formation in irradiated mice (**c** and **d**). The HE staining data further demonstrated that psoralen treatment provided protective effects on the bone structures of irradiated mice (**e**). The ELISA data showed that psoralen treatment reduces the TRAP level in serum of irradiated mice (**f**). Gene expression analysis of TRAF6 and OCN in femurs also suggested that psoralen inhibited osteoclastogenesis while favoring osteogenesis in irradiated mice (**g**). Further pathological analysis showed that psoralen partially restored the irradiation induced the reduction of OCN-labeled OBs and the increase of TRAP-labeled OCs in the irradiated mice (**h** and **i**). All data are shown as the mean ± SD. **P < 0.01, *P < 0.05. The scale bars represent 2 mm (**a** and **c**), 500 μm (**e**), and 200 μm (**h**), respectively. IR: irradiation; BV/TV: bone volume per tissue volume; Tb.N: trabecular bone number; Tb.Sp: trabecular separation; Tb.Th: trabecular bone thickness; BFR: bone formation rate; MAR: mineral deposition rate; TRAP: tartrate-resistant acid phosphatase; OCN: osteocalcin; TRAF6: TNF receptor-associated factor 6; Ob.S/B.S: osteoblast surface per bone surface; Ob.N/B.Pm: number of osteoblasts per bone perimeter; Oc.S/B.S: osteoclast surface per bone surface; and Oc.N/B.Pm: number of osteoclasts per bone perimeter
