# Supplementary figures and images for: Psoralen alleviates radiation-induced bone injury by rescuing skeletal stem cell stemness through AKT-mediated upregulation of GSK-3β and NRF2
Source: Stem Cell Res Ther. 2022 Jun 7;13:241. doi: 10.1186/s13287-022-02911-2 (PMC9172007; doi:10.1186/s13287-022-02911-2)

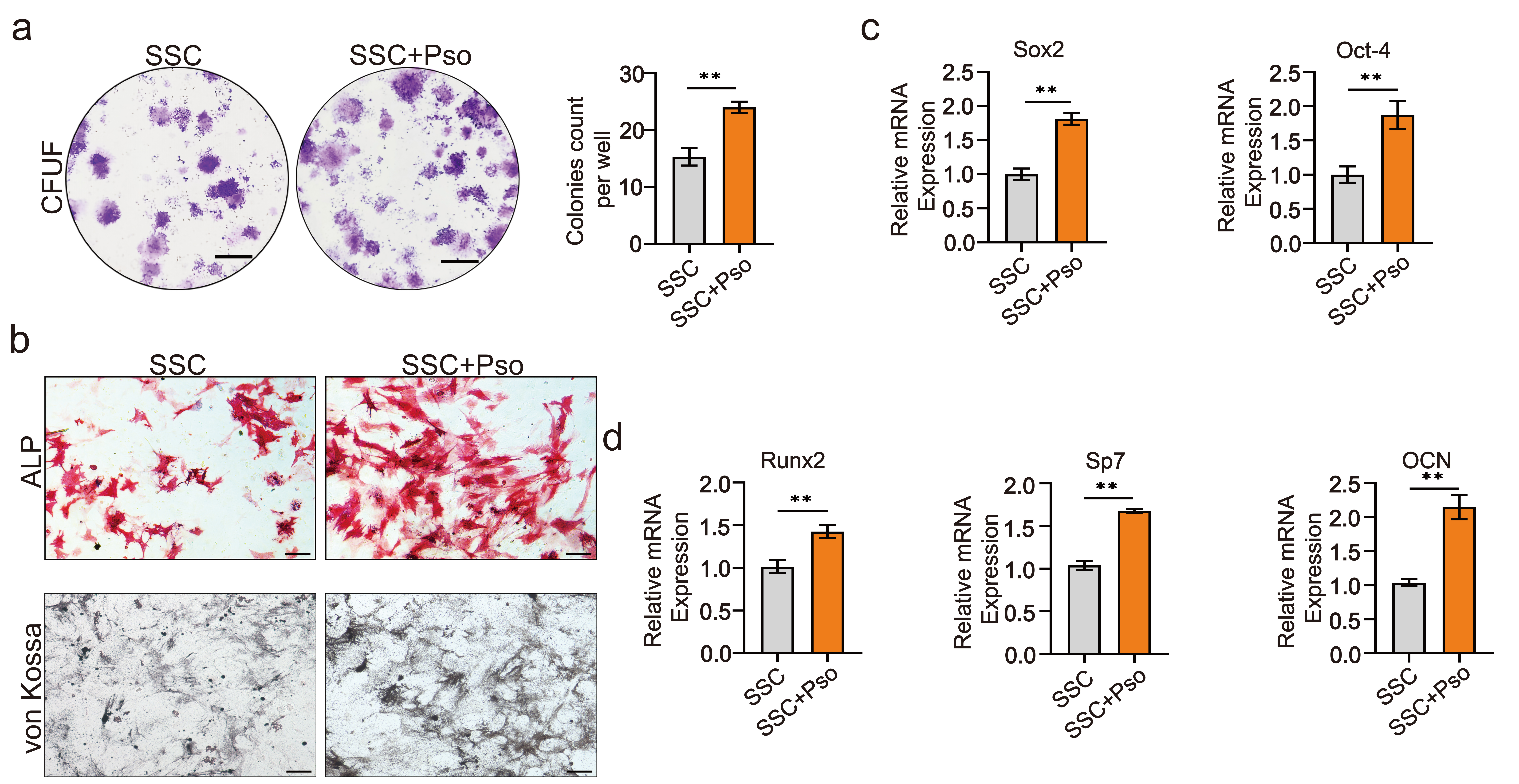

Supplement: Supplementary file 3 — Additional file 3. Figure S3. Psoralen promote the self-renewal and osteogenic differentiation of SSCs. The results of the CFU-F assay demonstrated that psoralen significantly promoted the cell colony formation of the SSC (Fig. S3a). In addition, the results of ALP staining and von Kossa staining showed that that psoralen promoted osteogenic differentiation of SSCs (Fig. S3b). The expression of self-renewal-related genes, including Sox2 and Oct-4, and osteogenesis-related genes, including Runx2, Sp7 and OCN, in SSCs was significantly upregulated in the presence of psoralen (Fig. S3c and S3d). [file 13287_2022_2911_MOESM3_ESM.jpg]

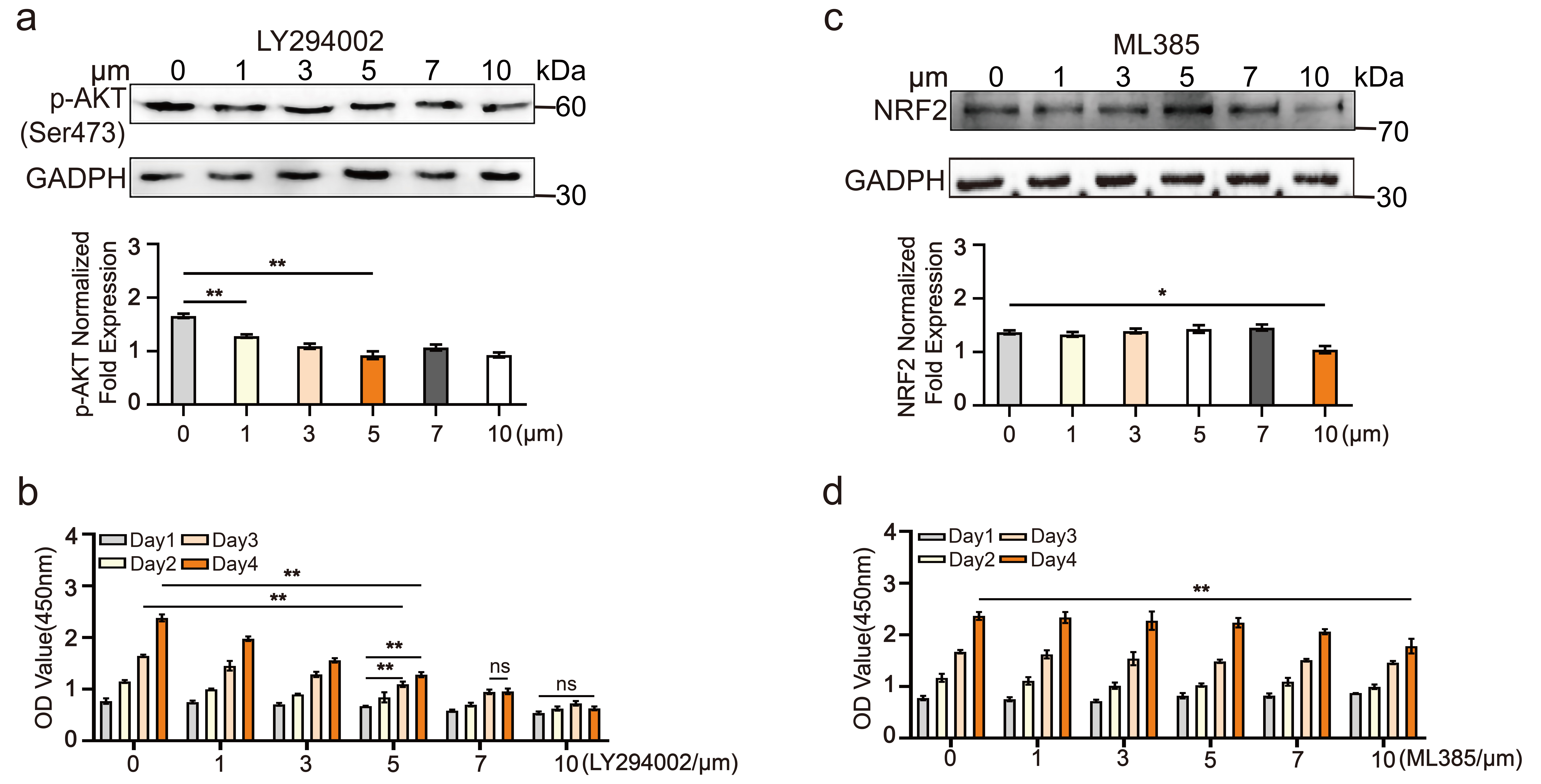

Supplement: Supplementary file 4 — Additional file 4. Figure S4. The screening of the appropriated concentration of LY294002 and ML385 for SSC proliferation and NRF2 expression in SSCs. The appropriate concentration of LY294002 was screened by using western blotting and CCK-8 assays (Fig. S4a and S4b). The appropriate concentration of ML385 was screened by using western blotting and CCK-8 assays (Fig. S4c and S4d). [file 13287_2022_2911_MOESM4_ESM.jpg]
