# Supplementary material for: Psoralen alleviates radiation-induced bone injury by rescuing skeletal stem cell stemness through AKT-mediated upregulation of GSK-3β and NRF2
Source: Stem Cell Res Ther. 2022 Jun 7;13:241. doi: 10.1186/s13287-022-02911-2 (PMC9172007; doi:10.1186/s13287-022-02911-2)
Supplement: Supplementary file 5 — Additional file 5. Supplementary Table 1. Primers used for RT-PCR analysis. [file 13287_2022_2911_MOESM5_ESM.docx]

**Supplementary Table 1 Primers used for RT-PCR analysis**

| Gene | Forward Primer (5’-3’) | Reverse Primer (5’-3’) |
| --- | --- | --- |
| Sox2 | CGGCACAGATGCAACCGAT | CCGTTCATGTAGGTCTGCG |
| Oct-4 | CGGGTTTCAACGCCGACTA | TTGGCACTAGAGACGGACAGA |
| TRAF6 | TACGATGTGGAGTTTGACCCA | CACTGCTTCCCGTAAAGCCAT |
| Runx2 | GACTGTGGTTACCGTCATGGC | ACTTGGTTTTTCATAACAGCGGA |
| Sp7/Osx | GGAAAGGAGGCACAAAGAAGC | CCCCTTAGGCACTAGGAGC |
| Bglap/OCN | GAACAGACAAGTCCCACACAGC | TCAGCAGAGTGAGCAGAAAGAT |
| Spp1 | TTCTGGCAGCTCAGAGGAGA | TTGACTCATGGCTGCCCTTT |
| Col1a1 | TAAGGGTCCCCAATGGTGAGA | GGGTCCCTCGACTCCTACAT |
| Mepe | AAATATCACGCAGCCTGTAAAGA | GCTGGAATTACGCTTAGAACACT |
| Dmp1 | CTGAAGAGAGGACGGGTGATT | CGTGTGGTCACTATTTGCCTG |
| Bmpr1b | CCCTCGGCCCAAGATCCTA | CAACAGGCATTCCAGAGTCATC |
| Gli2 | GGGACTCTTTAGCCTCGCAG | CCACAGGGTTGAGGTAGTCAT |
| Bmp8b | CCGGGACTCCTATGGCTACT | CATCCGTCATGGCACGGTA |
| GCLC | CTACCACGCAGTCAAGGACC | CCTCCATTCAGTAACAACTGGAC |
| GCLM | CTTCGCCTCCGATTGAAGATG | AAAGGCAGTCAAATCTGGTGG |
| NQO1 | AGAGAGTGCTCGTAGCAGGAT | GTGGTGATAGAAAGCAAGGTCTT |
| HOMX1 | AGGTACACATCCAAGCCGAGA | CATCACCAGCTTAAAGCCTTCT |
| GAPDH | CGGTGCTGAGTATGTCGTGGAGTCT | GCTAAGCAGTTGGTGGTGCAGGATG |
